# The dual role of cannabidiol on monocyte-derived dendritic cell differentiation and maturation

**DOI:** 10.3389/fimmu.2023.1240800

**Published:** 2023-08-22

**Authors:** Zsófia Pénzes, Shahrzad Alimohammadi, Dorottya Horváth, Attila Oláh, Balázs István Tóth, Attila Bácsi, Attila Gábor Szöllősi

**Affiliations:** ^1^ Department of Immunology, Faculty of Medicine, University of Debrecen, Debrecen, Hungary; ^2^ Doctoral School of Molecular Medicine, University of Debrecen, Debrecen, Hungary; ^3^ Department of Dermatology, School of Medicine, University of California, San Diego, La Jolla, CA, United States; ^4^ Department of Physiology, Faculty of Medicine, University of Debrecen, Debrecen, Hungary; ^5^ ELKH-DE Allergology Research Group, Debrecen, Hungary

**Keywords:** phytocannabinoids, cannabidiol, monocyte-derived dendritic cells, innate immunity, T cell proliferation

## Abstract

**Introduction:**

Extracts and compounds isolated from hemp (Cannabis sativa) are increasingly gaining popularity in the treatment of a number of diseases, with topical formulations for dermatological conditions leading the way. Phytocannabinoids such as ( )-cannabidiol, ( )-cannabinol and ( )-Δ9-tetrahydrocannabivarin (CBD, CBN, and THCV, respectively), are present in variable amounts in the plant, and have been shown to have mostly anti-inflammatory effects both in vitro and in vivo, albeit dominantly in murine models. The role of phytocannabinoids in regulating responses of dendritic cells (DCs) remains unclear.

**Methods:**

Our research aimed to investigate the effects of CBD, CBN, and THCV on human DCs differentiated from monocytes (moDCs). moDCs were treated with up to 10 μM of each phytocannabinoid, and their effects on viability, differentiation, and maturation were assessed both alone, and in conjunction with TLR agonists. The effects of CBD on cytokine production, T cell activation and polarization as well as the transcriptome of moDCs was also determined.

**Results:**

Phytocannabinoids did not influence the viability of moDCs up to 10 μM, and only CBD had effects on maturational markers of moDCs, and neither compound influenced LPS-induced activation at 10 μM. Since only CBD had measurable effects on moDCs, in our subsequent experiments we tested the effect only of that pCB. On moDCs differentiated in the presence of CBD subsequent activation by LPS induced a markedly different, much more tolerogenic response. CBD-treated moDCs also produced significantly more interleukin (IL)-6, TNFα and, importantly, IL-10 in response to LPS, which shows a shift toward anti-inflammatory signaling, as well as a more robust secretory response in general. To rule out the possibility that these effects of CBD are specific to TLR4 signaling, we determined the effect of CBD on TLR7/8-induced maturation as well, and saw similar, although less marked responses. CBD-treated moDCs were also less efficient at activating naïve T cells after LPS stimulation, further supporting the tolerogenic effect of this phytocannabinoid on moDCs. Reactome pathway analysis showed an inflammatory response to LPS in moDCs, and to a lesser extent to CBD as well. In contrast CBD-treated moDCs responded to LPS with a shift towards a more tolerogenic phenotype, as IL-10 signaling was the most prominently induced pathway in this group.

**Discussion:**

Our results show that CBD achieves an anti-inflammatory effect on adaptive immune responses only in the presence of an activating stimuli on moDCs by reprogramming cells during long-term treatment, and not through acute, short-term effects.

## 1 Introduction

Dendritic cells (DCs) are bone marrow-derived immune cells that are present in both secondary lymphoid organs and the periphery, including skin and mucosal tissues. DCs are a critical component of the immune response that couple innate and adaptive immunity, by acting as professional antigen-presenting cells to T cells and thereby initiating the adaptive immune response (1–3).

Immature DCs migrate to peripheral tissues where they continuously sample their environment until they encounter an activating stimulus. After activation they undergo a maturation process, during which the expression pattern of cell surface molecules (e.g., CCR7, CD83, CD86, DC-SIGN [CD209], HLA-DR), as well as the cytokines and inflammatory mediators they produce, changes. The maturation process takes place during migration into the lymph nodes, with the cells having a mature phenotype once they reach the lymph nodes. Mature DCs are essential for activation of naïve T cells, as they are the main antigen presenting cell population that initiates their proliferation and subsequent differentiation (4, 5).

The skin performs a complex physico-chemical and immunological function, forming a barrier against external pathogenic microorganisms by the cooperation of several antigen-presenting cells (Langerhans cells [LCs], dermal DCs), and is thus a key player in innate immunological defense. Dermal DCs are subtypes of antigen-presenting cells in the dermis and have a crucial role in cutaneous immune homeostasis (6, 7).

Under normal homeostatic conditions, dermal DCs can be divided into three main types: i) CD141^hi^ conventional type 1 dendritic cells (cDC1s); ii) CD1a^+^, CD1c^+^, and CD103^+^ cDC2s; and iii) CD1a^+^, CD1c^+^, and CD103^-^ monocyte-derived DCs (moDCs) (8). The functional role of these subgroups has not yet been fully characterized, and there is some debate regarding the plasticity of these cells, and their ability to alter their subset identity, especially in inflammatory settings (9). Traditionally cDC1 cells were thought to preferentially activate CD8^+^ T cells, while cDC2 and moDCs are considered potent inducers of CD4^+^ T cell proliferation (10, 11). Alterations in the balance of the three subsets of immune cells can be the starting point for the onset of chronic inflammatory skin diseases, as they can both lead to inflammation and promote tolerance in the skin. Inflammatory skin diseases affect many people, and their incidence has shown an increasing trend year-over-year (12–14).

moDCs are commonly used as a model to investigate prototypical cDC functions (15–18), and they can be readily found in any tissue during an inflammatory response (19–21). Their role in the steady state in most tissues is less well defined, as they were first thought to only appear at the onset of inflammation (19), and only more recent work has shown that they contribute to the renewal of cDC and LC populations to some extent (18, 22–25). Specifically in inflammatory skin diseases the role of moDCs has been proven in psoriasis, where they produce interleukin (IL)-1β and tumor necrosis factor (TNF) to amplify the inflammatory milieu, but are not solely responsible for it (26, 27). Newer data from psoriatic skin has also shown that myeloid DCs are the predominate antigen presenting cell in the psoriatic epidermis (28). In atopic dermatitis, a highly prevalent inflammatory skin disease which is characterized by Th2 dominant responses, moDCs found in lesional skin express FcϵRI and FcϵRII, and the number of recruited DCs increases with the development of clinically visible eczematous skin lesions. Th2 cytokines in the lesional skin direct the differentiation of recruited monocytes to moDCs, which can ultimately lead to the amplification of disease-specific Th cell responses *in vivo* (29). DC-SIGN, which is upregulated in DCs in the lesional skin of atopic dermatitis, can bind to a broad range of common allergens such as house dust mite allergen, egg white allergen, (30) and even transglutaminase 3, which has been proposed as an important autoallergen in atopic dermatitis, highlighting the important role of these cells in the pathogenesis of the disease (31). moDCs have also been investigated in systemic sclerosis, where single-cell RNA-Seq analysis showed that myeloid cells are abundant in diseased skin, and that moDC numbers correlated to disease severity (32), and that they can drive profibrotic inflammation and aberrant T cell polarization (33).

Known mainly for its psychotropic effects, hemp (*Cannabis sativa*) and the positive effects of the substances it produces are now increasingly being exploited in the treatment of a growing number of diseases, including the development of topical formulations for dermatological conditions. In the plant there are more than 100, mostly non-psychotropic compounds collectively termed phytocannabinoids (pCBs), e.g., (-)-cannabidiol (CBD), (-)-cannabinol (CBN), and (-)-Δ^9^-tetrahydrocannabivarin (THCV). It is widely accepted that pCBs have mainly anti-inflammatory properties, but their mechanism of action is not well understood (34–36). Specifically, our understanding of cannabinoid-based DC biology, and especially of the effects of pCBs, is greatly lacking. Of the few studies that have investigated the beneficial effects of pCBs in inflammatory skin diseases, much of the published literature is from mouse models (37, 38). Since human and mouse skin show substantial differences in both structure and immunology (39), it is essential to investigate the potential impacts of pCBs on human DCs.

Specifically on human DCs, only the effect of the most important psychotropic pCB, (-)-*trans*-Δ^9^-tetrahydrocannabinol (THC) has been investigated. It was reported that THC inhibited moDC differentiation and T cell activation, and altered the expression of CD11c, HLA-DR, as well as of certain costimulatory molecules (CD40 and CD86). Moreover, it was found to suppress IL-12 cytokine production as well; thus, it may also have an impact on anti-microbial immune responses (40).

In the context of inflammatory skin disease, CBD-enriched ointment treatment seems safe for certain skin disorders. In 2019, Palmieri et al. investigated the effect of CBD-enriched ointment on different inflammatory skin diseases, cutaneous scars, and wound healing. In this pilot study they examined 20 patients, who suffered from chronic skin disorders (psoriasis, atopic dermatitis, and resulting outcome scars) and used a CBD-enriched ointment twice daily for three months. Topical administration of CBD-enriched ointment resulted in improved skin parameters (hydration, transepidermal water loss, elasticity) and marked improvements in the quality of life of the patients (41). Similar effects were reported with the use of topical retinol and water-soluble CBD (42).

Topical pCBs have been investigated in three clinical trials for the treatment of acne, atopic dermatitis and epidermolysis bullosa [(43–45) respectively]. CBD proved to be ineffective in atopic dermatitis, but showed more promising results in certain acne patients, while the investigation of CBN on epidermolysis bullosa is still ongoing. Topical pCBs were also reported to be effective in certain types of chronic wounds, such as venous leg ulcers, non-uremic calciphylaxis, and pyoderma gangrenosum (46–48).

In mice, topical skin application also proved the anti-inflammatory effect of CBD and palmitoylethanolamide (an endogenous cannabinoid-related substance) in a 12-O-tetradecanoylphorbol-13-acetate-induced dermatitis model. Use of the topical CBD treatment significantly decreased ear edema by 51.27% at 24 hours and 65.69% at 48 hours post utilization compared to the baseline, and negative control group, supporting its anti-inflammatory capability (49).

Recent studies have reinforced the anti-inflammatory potential of CBD on human primary leukocytes. Blevins et al. screened the effect of pCBs (CBD, (-)-cannabigerol, (-)-cannabidivarin, and THC) on human peripheral blood monomorphonuclear cells (PBMCs), and analyzed the effect of pCB-pretreatment on CpG (plasmacytoid DC), lipopolysaccharide (LPS) (monocytes), or anti-CD3/CD28 (T cells) activating stimuli. In this way, they could assess the proliferation, activation marker expression, cytokine expression, cytokine production and phagocytosis of the cells (where relevant). All cannabinoids (including THC) suppressed the secretion of proinflammatory cytokines (e.g., IL-1β, IL-6, TNFα) to some degree, as well as the phagocytotic activity of monocyte-derived CD14^+^ cells (50). Similar effects for DCs have not yet been described.

Thus, the effects of pCBs on human moDCs have significant potential. Because of the described anti-inflammatory activity of cannabinoids in the treatment of many immune-mediated skin diseases, they may also have a positive dermatological impact on certain aspects of e.g., acne, atopic dermatitis, or psoriasis (36).

Therefore, our research aimed to investigate the effects of selected non-psychotropic pCBs (CBD, CBN, and THCV) on human DCs in regulating responses of moDCs, an accepted model of DC biology.

## 2 Materials and methods

### 2.1 Reagents and antibodies

CBD and THCV were purchased from ChemFaces (Wuhan, Hubei, PRC), while CBN was purchased from Cayman Chemical (Ann Arbor, MI). All pCBs were diluted in absolute ethanol. The final concentration of absolute ethanol in the culture medium was 1:1000.

Fetal Bovine Serum (FBS, Gibco™), and cell culture media (RPMI1640) were purchased from ThermoFisher Scientific (Waltham, MA, USA). IL-6, IL-10, IL-12, CXCL8 and TNFα ELISA kits were all from BD-Biosciences (San Jose, CA, USA). LPS and CL075 was obtained from Invivogen (San Diego, CA, USA) and dissolved in nuclease-free water.

Fluorescently labeled monoclonal antibodies (mAbs) against DC-SIGN (RRID: AB_1134045), CD83 (RRID: AB_314514), CD1a (RRID: AB_314020), CD207 (RRID: AB_2561590), CD4 (RRID : AB_571945) were sourced from BioLegend (San Diego, CA, USA), HLA-DQ (RRID: AB_2573320) mAbs were obtained from ThermoFisher Scientific, while CD86 (RRID: AB_2275742), IL4 (RRID : AB_357279) and CCR7 (RRID: AB_2259847) were from R&D Systems (Minneapolis, MI, USA), and CD3 (RRID : AB_395736) was from BD-Biosciences.

### 2.2 Analysis of cell viability

Cell viability was assessed by CyQUANT™ Cytotoxicity Assay Kit (which measures glucose-6-phosphate dehydrogenase [G6PD] release; λ_ex_ = 560 nm and λ_em_ = 590 nm) and PrestoBlue Cell Viability Reagent (ThermoFisher, λ_ex_ = 560 nm and λ_em_ = 590 nm). Cells were cultured in 96-well black-well/clear-bottom plates (Greiner Bio-One, Kremsmünster, Austria) in quadruplicates and were treated with various concentrations of pCBs over the course of their differentiation. In the case of G6PD release the supernatant was moved to a new plate, while PrestoBlue assay was performed on the remaining cells. In both assays we followed the manufacturer’s instructions. Fluorescence was detected using an EnVision 2105 Multimode Plate Reader (Perkin Elmer, Waltham, MA, USA).

### 2.3 Isolation of monocytes and differentiation of moDCs

Heparinized leukocyte-enriched buffy coats from human subjects were gathered from healthy individuals. The human samples used in this study were acquired from a by- product of routine care or industry. Written informed consent for participation was not required from the participants or the participants’ legal guardians/next of kin in accordance with the national legislation and institutional requirements. The process was approved by the Regional Blood Center of the Hungarian National Blood Transfusion Service (Debrecen, Hungary), with the written approval of both the Head of the National Transfusion Service and the Regional and Institutional Ethics Committee of the Faculty of Medicine at the University of Debrecen (Debrecen, Hungary; approval number: OVSZK 3572-2/2015/5200).

Peripheral blood monomorphonuclear cells (PBMCs) were extracted from buffy coats using Ficoll gradient centrifugation. Monocytes were obtained from PBMCs with the help of special immunomagnetic beads linked to anti-CD14 antibodies (Miltenyi Biotec, Bergish Gladbach, Germany), following the manufacturer’s directions.

Primary human moDCs were differentiated from monocytes using established protocols (51). Briefly, monocytes were cultured in 24-well plates in RPMI 1640 medium (Sigma-Aldrich, St. Louis, MI, USA) containing 10% heat-inactivated FBS, 10 mM HEPES, 100 μg/ml penicillin/streptomycin, 50 mM 2-mercaptoethanol (all from Sigma-Aldrich), GM-CSF (80 ng/ml) (Gentaur Molecular Products, London, UK) and IL-4 (20 ng/ml) (PeproTech, Brussels, Belgium) at a density of 1 × 10^6^ cells/ml for 6 days. On day 3 of culturing half of the culture medium was removed and replaced by fresh medium containing the full cytokine amount. As a positive control of maturation LPS (250 ng/ml) was applied for 24 hours on day 4.

The effect of pCBs on the differentiation process was assessed by adding pCBs (10 µM CBD, CBN, or THCV) or the vehicle (absolute ethanol) alone on days 0 and 3 of the protocol and determining the expression of DC-SIGN/CD209 and CD14. Expression of maturation markers (CD1a, CCR7, CD83, CD86, HLA-DQ) was determined on day 5 of the culturing protocol with fluorescence-activated cell sorting (FACS) using the 2000R Novocyte Flow cytometer (ACEA Biosciences, San Diego, CA, USA).

### 2.4 Isolation of naïve CD4^+^ T cells and T cell proliferation assay

For mixed leucocyte reaction (MLR) human naïve CD4^+^ T cells were separated by negative selection from PBMC using a human Naïve CD4^+^ Isolation Kit (Miltenyi Biotec) according to the manufacturer’s protocol.

For the proliferation assay, naïve T cells were labeled with 0.5 µM carboxyfluorescein succinimidyl ester (CFSE, ThermoFisher Scientific). moDCs were cultured with 2 × 10^5^ naïve T cells at 1:10 moDC:naïve T cell ratios in 96 well round bottom cell culture plates in 200 μl RPMI 1640 medium for 5 days. The co-cultures were supplemented with 1 μg/ml anti-human CD3 mAb (BD-Biosciences). T cells were collected on day 5, fluorescence intensities were measured on FL1 (530 ± 15 nm) channel with FACS by Novocyte 2000R flow cytometer and data were analyzed with FlowJo 10.8.1 software (FlowJo, Ashland, OR, USA).

### 2.5 Intracellular IL-4 cytokine staining/cell polarization and ELISpot assay

To perform intracellular cytokine staining, naïve CD4^+^ T cells were plated into 96 well round bottom cell culture plates in 200 μl RPMI 1640 medium for 5 days with moDCs differentiated as described above at a ratio of 1:10 (2 × 10^5^/ml moDCs; 2 × 10^6^/ml naïve T cells). After 5 days of coculturing, the cells were stimulated with 50 ng/ml phorbol myristate acetate (PMA) and 1 μg/ml ionomycin (all from Sigma-Aldrich) for 4 hours, followed by the presence of the protein transport inhibitor monensin (BD-Biosciences). Subsequently, the cells were first stained with anti-CD4-APC (extracellular staining), followed by fixation and permeabilization using the BD Cytofix/Cytoperm solution (BD-Biosciences). The cells were then labeled with anti-CD4-APC and anti-IL-4-PE antibodies (intracellular staining). Fluorescence intensities were measured using a Novocyte 2000R flow cytometer (ACEA Biosciences). Results were evaluated using FlowJo 10.8.1 software (FlowJo).

T cells for the ELISpot assay (Mabtech, Nacka Strand, Sweden) were seeded at the same cell ratio as described in the T cell polarization assay. After 5 days of coculturing, the cells were washed and reseeded on an ELISpot plate coated with 0.5 μg/ml mouse anti-human CD3 antibody for 40 hours. The Th2 responses were analyzed by avidin-horseradish peroxidase-based enzyme-linked ImmunoSpot (ELISpot) system using an ImmunoScan plate reader (Cell Technology Limited, Hong Kong, Kowloon, SAR).

### 2.6 Flow cytometry analysis

Staining was performed in phosphate-buffered saline (PBS) buffer containing 2 (v/v) % heat-inactivated FBS and 2 mM EDTA (pH 7.4). Cells were stained on ice for 20 min, washed twice with PBS-based buffer, resuspended in 100 μl buffer, and kept on ice until measurement.

Measurements were performed with a Novocyte 2000R flow cytometer (ACEA Biosciences). Results were evaluated using FlowJo 10.8.1 software (FlowJo).

### 2.7 Enzyme-linked immunosorbent assay (ELISA)

Determination of cytokine levels from primary moDCs was performed using ELISA. Supernatants were collected in the case of immature moDCs on day 5 and for the matured cells on day 6. IL-6, -8, -10, -12, and TNFα ELISA kits were all from BD-Biosciences, and determination was performed according to the manufacturer’s instructions. The cytokine levels were measured by EnVision 2105 Multimode Plate Reader (Perkin Elmer). The concentration of the cytokines were then calculated with cubic logistic model (52) using GraphPad Prism 9.1.2 for Windows (GraphPad Software Inc., La Jolla, CA, USA).

### 2.8 RNA-sequencing (RNA-Seq)

High throughput mRNA sequencing analysis was performed on an Illumina sequencing platform. Samples were collected on day 6 of differentiation protocols. Agilent BioAnalyzer with Eukaryotic Total RNA Nano Kit (Agilent Technologies, Waldbronn, Germany) was used for checking RNA integrity (RIN). RNA samples with integrity number >7 were accepted for the library preparation process. mRNA-Seq libraries were prepared from total RNA using Ultra II RNA Sample Prep kit (New England BioLabs Inc., Ipswich, MA, USA) according to the manufacturer’s protocol. Sequencing runs were performed on Illumina NextSeq 500 instrument using single-end 75 cycle sequencing. HISAT2 algorithm was used for alignment of raw sequencing reads to human reference genome version GRCh38. StrandNGS software (www.strand-ngs.com) was used for further statistical analysis. Aligned data were normalized by using DESeq2 algorithm (53). Library preparations, sequencing, and primary data analysis were performed at Genomic Medicine and Bioinformatics Core Facility of the University of Debrecen, Hungary. Analysis was also performed using the Galaxy web platform, through the public server at usegalaxy.org (54). Functional enrichment analysis of the differentially expressed genes was conducted using gProfiler (https://biit.cs.ut.ee/gprofiler/). The gene list was uploaded to the gProfiler web interface, and enrichment analysis was performed against the Reactome pathways database. Significantly enriched terms with an adjusted p-value < 0.05 were considered biologically relevant. Network analysis was performed using Cytoscape 3.9.1 (https://cytoscape.org/), and the network was visualized and analyzed using the EnrichmentMap Cytoscape plugin (55–58).

### 2.9 Statistical analysis

Statistical evaluation was done through GraphPad Prism 9.1.2. for Windows (GraphPad Software Inc.). Two-tailed, unpaired Student’s t-test was used to compare two groups while one-way ANOVA was used to assess groups of three or more followed by Tukey’s or Dunnett’s test. Differences were considered to be statistically significant at P < 0.05, unless otherwise stated.

## 3 Results

### 3.1 pCBs do not influence cellular viability, and only CBD influences maturation marker expression of moDCs

**Figure 1:**
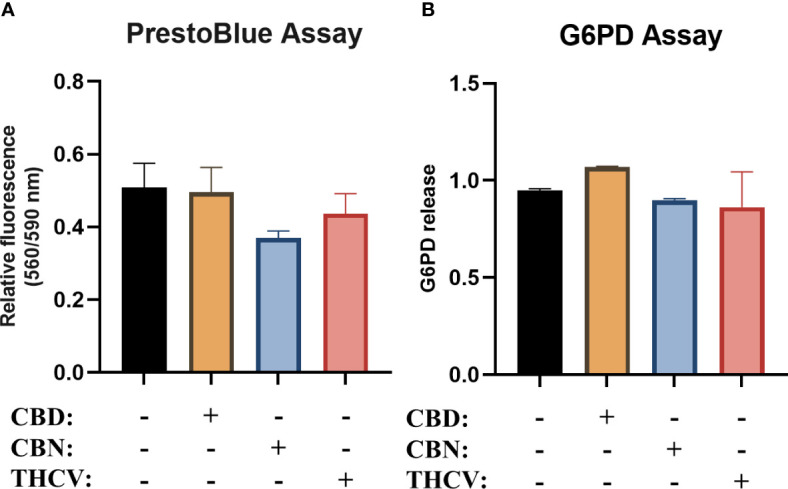
Non-psychotropic pCBs do not decrease viability of moDCs. Monocytes were cultured in the presence of GM-CSF and IL-4 for five days to generate moDCs in the presence of 10 µM CBD, CBN, THCV or vehicle (0.1 v/v% absolute ethanol). Determination of moDCs viability by PrestoBlue Assay **(A)** and glucose-6-phosphate dehydrogenase (G6PD) release assay **(B)**. N≥3, mean ± SD. CBD: (-)-cannabidiol, CBN: (-)-cannabinol, THCV: (-)-Δ^9^-tetrahydrocannabivarin.

As a first step in our experiments, we performed viability studies to determine the optimal non-toxic dose of the selected non-psychotropic pCBs (CBD, CBN, and THCV) in regulating responses of moDCs. After differentiation of the moDCs, we determined the viability of the cells using PrestoBlue, and glucose-6-phosphate dehydrogenase (G6PD) release as a marker of necrotic cell death from the cell supernatant. pCBs did not induce any significant cell death in either assay (**Figures 1A, B**).

**Figure 2:**
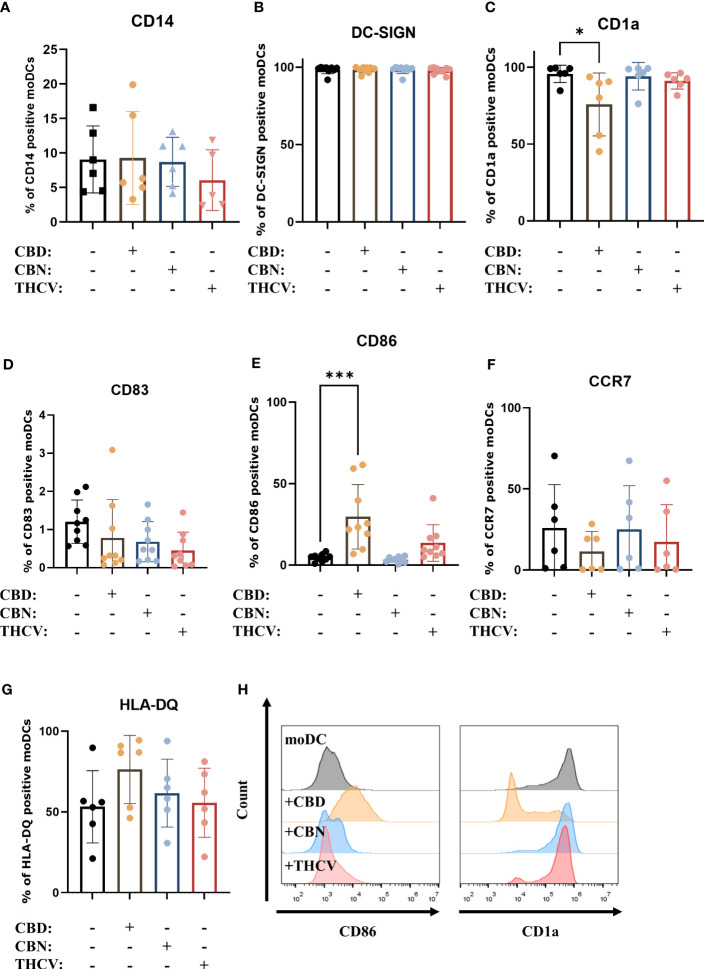
Non-psychotropic pCBs do not influence differentiation or maturation of moDCs. Monocytes were cultured in the presence of GM-CSF and IL-4 for five days to generate moDCs in the presence of 10 µM CBD, CBN, THCV or vehicle (0.1 v/v% absolute ethanol). Percentage of cells positive for CD14 **(A)**, DC-SIGN **(B)**, CD1a **(C)**, CD83 **(D)**, CD86 **(E)** CCR7 **(F)** and HLA-DQ **(G)** following the indicated treatments. N=6-9 donors, mean ± SD, *p < 0.05, ***p < 0.001, as indicated (determined by repeated measures one-way ANOVA). Individual donors are represented by symbols. **(H)** Representative histograms of significant changes from previous panels showing CD86 (left) and CD1a expression (right). moDC: vehicle-treated monocyte-derived dendritic cell, CBD: (-)-cannabidiol, CBN: (-)-cannabinol, THCV: (-)-Δ^9^-tetrahydrocannabivarin.

Next, we investigated the effect of the pCBs (i.e., CBD, CBN, and THCV), on the differentiation and maturation of moDCs. To assess the differentiation of the cells we examined the expression of CD14, CD1a and DC-SIGN (**Figures 2A–C**), while we determined the maturational state of moDCs by investigating the expression of HLA-DQ, costimulatory markers CD86 and CD83, the chemokine receptor CCR7 (**Figures 2D–H**). These markers were also assessed on monocytes, where we determined high CD14, low CD1a and DC-SIGN positivity, as well as negligible HLA-DQ, CD83 and CCR7 expression, while CD86 was present on approximately 30% of monocytes as shown on **Supplementary Figure 1**. As expected by day 5 the expression of CD14 was on average below 10% in all groups, while almost all cells were DC-SIGN positive. As CD1a is commonly expressed on moDCs we also investigated its expression and found that CBD decreased the percentage of positive cells. Of the investigated pCBs, only CBD had some effect on maturational markers, since it increased the expression of CD86 and HLA-DQ, and decreased the ratio of CCR7 positive cells (although the latter two effects did not reach the level of statistical significance). These data suggest that neither pCB influences the differentiation of moDCs to a significant degree, and only CBD has effects on their maturation. An increase in CD86 and HLA-DQ expression suggests a proinflammatory response, but this is undercut by the drop in CCR7, which hints that the cells are less likely to migrate to draining lymph nodes. Since CD1a is expressed on both immature and mature moDCs (59, 60), the significant decrease in CD1a expression in CBD-treated cells does not signal a move towards either immature or mature states, but rather a more subtle impact on the development of the cells. The increase in CD86 and decrease in CCR7 expression in CBD-treated cells showed the same trend among all investigated donors, while the effect on HLA-DQ expression was more varied, as shown by pairwise comparisons in **Supplementary Figure 2**. Representative histograms of statistically significant changes are shown in **Figure 2H**.

### 3.2 CBD does not inhibit TLR agonist-induced activation of moDCs

**Figure 3:**
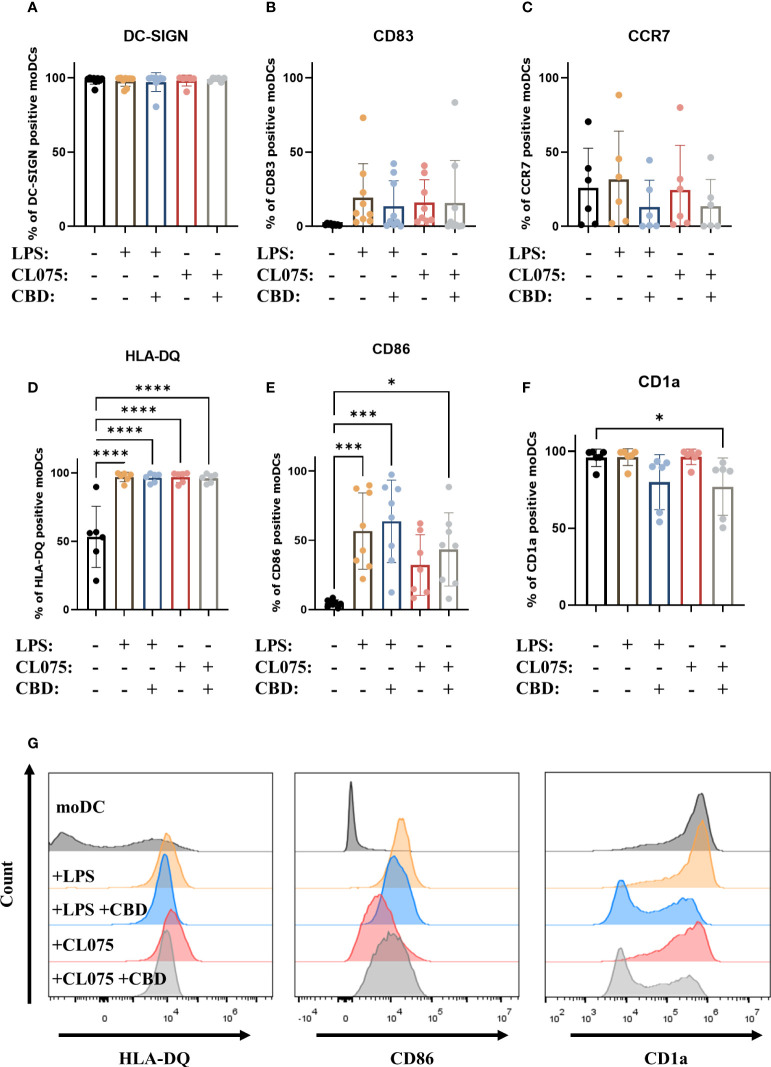
CBD does not influence LPS and CL075-induced maturation. Monocytes were cultured in the presence of GM-CSF and IL-4 for five days to generate moDCs in the presence 10 µM CBD or vehicle (0.1 v/v% absolute ethanol). Maturation was induced by lipopolysaccharide (LPS: 250 ng/ml) and TLR7/8 activation (CL075: 500 ng/ml) for 24 hours started on day 5. Percentage of cells positive for DC-SIGN **(A)**, CD83 **(B)**, CCR7 **(C)**, HLA-DQ **(D)**, CD86 **(E)**, and CD1a **(F)** are shown. N=6-8 donors, mean ± SD, *p < 0.05, ***p < 0.001, ****p < 0.0001 by repeated measures one-way ANOVA, as indicated. Individual donors are represented by symbols. **(G)** Representative histograms of significant changes from previous panels showing HLA-DQ (left), CD86 (middle) and CD1a expression (right). moDC, vehicle-treated monocyte-derived dendritic cell; CBD: (-)-cannabidiol, CL075, TLR7/8 agonist; LPS, lipopolysaccharide.

Since only CBD had measurable effects on moDCs, in our subsequent experiments we investigated the impact of this pCB on maturation processes induced by Toll-like receptor (TLR) agonists lipopolysaccharide (LPS) and CL075 (an agonist of TLR4 and TLR7/8, respectively, 61, 62). Neither TLR agonist was sufficient to elicit maturation of moDCs alone, as evidenced by the high percentage of DC-SIGN positive cells, and the fact that the increase in CD83 and CCR7 expression did not reach statistical significance (**Figures 3A–C**). Both TLR agonists caused a significant increase in the ratio of HLA-DQ positive cells, and LPS could also induce CD86 expression. Although all cells were HLA-DQ positive, there were marked differences in the mean fluorescence intensity (MFI) of the cells, highlighting the variance of protein expression between donors. Both LPS and CL075 significantly increased the MFI of HLA-DQ on activated DCs, while the cells differentiated in the presence of CBD showed less marked increases, which was not statistically significant compared to the moDC group (**Supplementary Figure 3**). CL075 alone was insufficient to increase CD86 expression, but the addition of CBD resulted in a higher percentage of positive cells, supporting the pro- rather than anti-inflammatory effect of CBD in this instance (**Figures 3D, E**). The decrease in CD1a positive cells was also present in CBD-treated groups, although it did not reach statistical significance in LPS and CBD-treated cells (**Figure 3F**). Representative histograms of statistically significant changes are shown in **Figure 3G**.

### 3.3 CBD increases cytokine secretion of human moDCs

**Figure 4:**
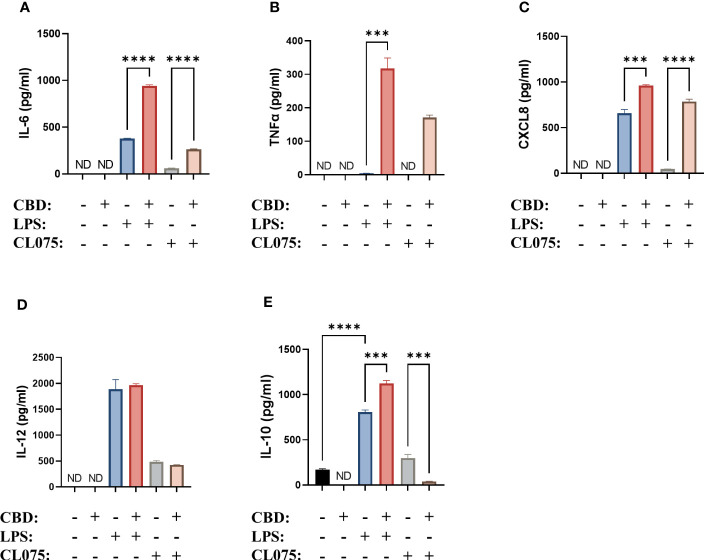
CBD can further increase cytokine production induced by TLR4 and TLR7/8 activation on moDCs. Monocytes were cultured in the presence of GM-CSF and IL-4 for five days to generate moDCs in the presence of 10 µM CBD or vehicle (0.1 v/v% absolute ethanol). Maturation was induced by applying lipopolysaccharide (LPS: 250 ng/ml) or TLR7/8 agonist (CL075: 500 ng/ml) for 24 h on day 5. IL-6 **(A)**, TNFα **(B)**, CXCL8 **(C)**, IL-12 **(D)** and IL-10 **(E)** production was determined with ELISA from supernatants. Bar plots represent mean ± SD of representative results from N≥3 independent experiments, ***p < 0.001, ****p<0.0001 compared to the marked groups as determined by repeated measures one-way ANOVA. ND, not determined. CBD, (-)-cannabidiol; CL075, TLR7/8 agonist; LPS, lipopolysaccharide.

We next investigated whether the secretion of cytokines was influenced by CBD. We found that CBD alone did not induce the production of either proinflammatory cytokines (IL-6, TNFα, CXCL8, IL-12) or anti-inflammatory IL-10. Interestingly, when moDCs differentiated in the presence of CBD were activated with LPS, a significant increase in the secretion of IL-6, TNFα, CXCL8 and IL-10 could be measured (**Figures 4A–E**). Importantly, CBD applied acutely, i.e. to normally differentiated moDCs 10 minutes before activation with LPS had differing effects. Acute CBD treatment alone did not induce any cytokine secretion, and resulted in lower IL-6, higher IL-10 and TNFα secretion when applied with LPS (**Supplementary Figure 4**).

Similar to what we described in the case of the maturation markers, TLR7/8 agonism was less effective at inducing the production of the aforementioned cytokines. The addition of CBD once again further increased the production of IL-6, TNFα (which was not induced by CL075 alone), and CXCL8 (**Figures 4A–D**). Interestingly, the production of IL-10 was decreased instead of increased in this system (**Figure 4E**). It is also important to note that the overall amount of cytokines produced after CL075 treatment was much less than what we observed when using LPS.

### 3.4 CBD shifts the immune responses of human moDCs to a more tolerogenic state

**Figure 5:**
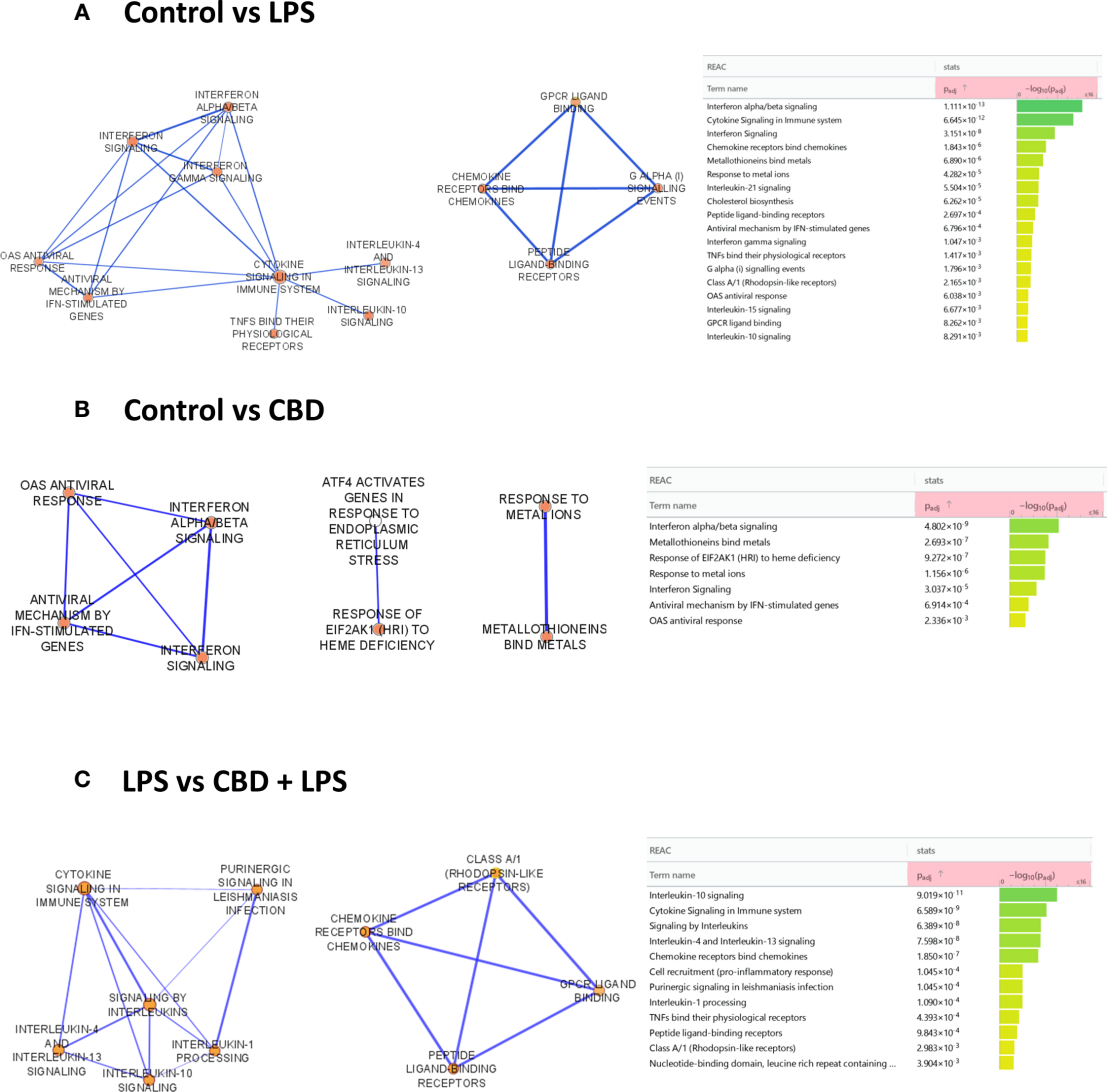
RNA-seq and Gene Ontology (GO) analysis revealed that CBD induced important alterations in several relevant signaling pathway of moDCs. GO classifications of the differentially expressed genes (DEGs) in LPS- and CBD- as well as LPS+CBD-treated moDCs based on reactome pathways. **(A)** Upregulated pathways in LPS-treated cells (mature moDCs) compared to vehicle-treated (immature) control moDCs. **(B)** Upregulated pathways in CBD-treated cells compared to vehicle-treated (immature) control moDCs. **(C)** Upregulated pathways in CBD and LPS-treated (mature) moDCs compared to LPS-treated (mature) moDCs. Nominal p values are shown to the right of the graphs. moDC, monocyte-derived dendritic cell; CBD, (-)-cannabidiol (10 μM); LPS, lipopolysaccharide (250 ng/ml).

To determine which molecular pathways are influenced in CBD-treated cells, we performed RNA-Seq analysis. Comparison of the control moDC gene set to DCs that underwent LPS-induced activation showed a dominantly proinflammatory response based on the enriched reactome pathways (58). The most significantly induced pathways in this comparison include “interferon alpha/beta signaling”, “chemokine receptors bind chemokines”, “metallothioneins bind metals”, “IL-10 signaling”, “OAS antiviral response, and towards the least significant pathways “IL-4 and IL-13 signaling” (**Figure 5A**, left panel). There were similarities when comparing the CBD-treated cells to the control group, with “interferon alpha/beta signaling”, “metallothioneins bind metals”, and “OAS antiviral response”, all occurring in both groups, albeit CBD caused a much less significant change than LPS did as shown by the lower adjusted p value in the latter comparison (**Figure 5B**). Interestingly, combined CBD-LPS treatment resulted in a marked shift in the induced pathways, with “IL-10 signaling” rising to clear prominence as the most significantly upregulated pathway, followed by the previously mentioned “chemokine receptors bind chemokines”, “IL-4 and IL-13 signaling”, and further down the list “IL-1 processing”. This shows that CBD is not necessarily anti-inflammatory when applied alone, since it initiates similar pathways to LPS treatment. When combined with a proinflammatory stimuli such as LPS however, CBD shifts the phenotype of DCs to a putatively more tolerogenic state, with increased production of the anti-inflammatory IL-10, and the induction of “IL-4 and IL-13 associated signaling”, which would facilitate Th2 T cell responses instead of more classically inflammatory Th1 or Th17 (**Figure 5C**).

### 3.5 CBD limits the T cell stimulating capability of moDCs

**Figure 6:**
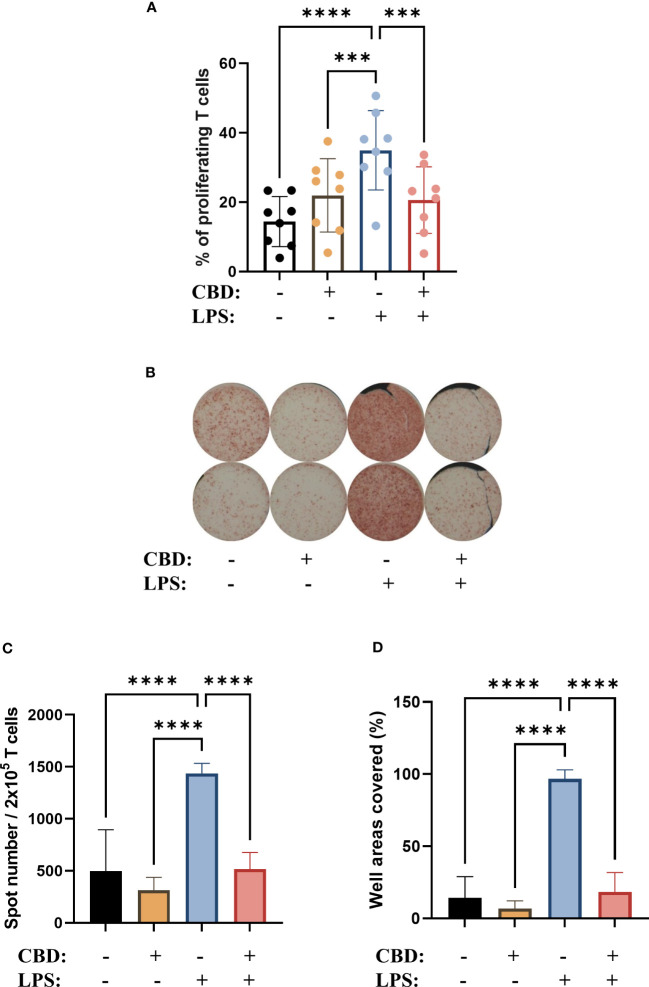
CBD-treated, LPS stimulated moDCs were less effective at inducing the proliferation of naïve T cells, and IL-4 polarizing cell capacity is also abrogated. Monocytes were cultured in the presence of GM-CSF and IL-4 for five days to generate immature moDCs in the presence of 10 µM concentration of CBD or vehicle (0.1 v/v% absolute ethanol). Maturation was induced by applying lipopolysaccharide (LPS: 250 ng/ml) treatment for 24 h on day 5. **(A)** Percentage of proliferating T cells after 5 days of coculture of naïve T cells and moDCs at a ratio of 10:1. N=8, mean ± SD, individual donors are represented by symbols. ***p<0.001, ****p < 0.0001, as determined by repeated measures one-way ANOVA compared to the marked groups. **(B)** Representative ELISpot image showing the quantity of IL-4 producing T cells in brown. **(C, D)** The average number of spots reflecting T cell responses were tallied from 5-6 technical repeats. The mean values and the converted well area of spot numbers were counted based on 2 independent donors. Bar plots represent mean ± SD. ****p<0.0001 compared to the marked groups as determined by repeated measures one-way ANOVA. CBD, (-)-cannabidiol; LPS, lipopolysaccharide.

As one of the most important links between the innate and adaptive arms of the immune system a cornerstone of DC function is their ability to induce T cell responses by activating naïve T cells. Investigating this important function we found that CBD applied alone throughout the differentiation of moDCs – but not to the T cell-moDC cocultures directly – did not influence the T cell stimulating capacity of these cells; however, the increase after LPS-induced maturation was limited in the CBD-treated group (**Figure 6A**, **Supplementary Figure 5**). CBD had no such effect when applied only at the same time as LPS (i.e., for 24 hours on day 5, data not shown), which hints that chronic exposure to this pCB reprograms the cells to be less responsive to this inflammatory stimulus.

The polarization of T cells induced by CBD-treated moDCs did not follow this trend, as when we investigated the ratio of IL-4^+^ cells among the activated T cells with intracellular cytokine staining (**Supplementary Figure 6**) and ELISpot (**Figures 6B–D**) we found that moDCs differentiated in the presence of CBD did not induce Th2 polarization, but did abrogate the increase in IL-4^+^ caused by LPS stimulation.

## 4 Discussion

In the last two decades there has been a rapid shift in the legal status of cannabis, and as a consequence, pCBs are increasingly used in commercially available extracts made from the plant, especially in topical formulations (50, 63, 64). The use of pCB-containing treatments has been bolstered by recent research in murine and canine models that highlights the anti-inflammatory potential of both pure compounds and mixed extracts.

Both THC and CBD could dose-dependently decrease the Th17 inflammatory autoimmune phenotype, which was tested by myelin oligodendrocyte glycoprotein (MOG_35-55_) of MOG_35-55_-specific encephalitogenic T cells in mice. The protein and mRNA expression of IL-6 were suppressed, while pretreatment with CBD elevated the levels of IL-10 (65).

Massimini et al. used polyphenols and CBD in a new *in vitro* model for canine atopic dermatitis. They found that both can modulate transcriptional regulation of Th1/Th2 inflammatory genes. The compound mixture induced in the inflamed keratinocytes a significant downregulation of the gene expression of CCL2, CCL17, and TSLP, and a similar downregulation of CCL2, CC17, IL31RA in monocytes (66).

While there is increasing evidence, especially in animal models as detailed above, that non-psychotropic pCBs (primarily CBD) are effective at reducing inflammation, we know significantly less about their effect on human cells. Research into non-psychotropic pCBs has increased markedly in the past two decades, driven in part by their use as anti-cancer treatments. These pCBs can indeed be cytotoxic at high doses (>15 μM), especially to cancerous cells (67), however CBD and CBN did not influence the viability of human PBMCs up to 10 μM (50). Other results show that pCBs can be cytotoxic, as 4-8 μM of CBD could induce apoptosis in mouse thymocytes (68), and primary monocytic cells were affected by 1-16 μM (69, 70). Therefore, to rule out any cytotoxic effect we first determined that CBD, CBN, and THCV did not induce cell death in moDCs (**Figures 1A, B**). Using these non-cytotoxic concentrations of pCBs we next investigated their effect on the differentiation and maturation of moDCs. Previous results on monocytes have shown that maturation marker expression induced by LPS stimulation is only minimally impacted by pCB pretreatment, specifically by (-)-cannabidivarin, but not by CBD or CBN (50). Partly in line with these results, in moDCs we found that only CBD decreased the percentage of CD1a positive cells, but caused no other changes in differentiation markers (**Figures 2A–C**) and did not influence LPS or CL075 induced changes with the exception of bolstering CD86 expression of the latter group (**Figure 3**). Similar to CBD none of the other pCBs impacted LPS-induced activation (**Supplementary Figure 7**).

In contrast to the minimal effects on differentiation and maturation markers moDCs differentiated in the presence of CBD showed increased IL-6, TNFα, and CXCL8 production, albeit only in the presence of LPS or CL075 (**Figure 4**). Acute CBD treatment had similar, albeit distinct effects, since it resulted in lower IL-6, higher IL-10 and TNFα secretion when applied with LPS (**Supplementary Figure 4**). Increased IL-10 production might be context-dependent, since it only occurs with the former but not the latter activating agent. CBD has recently been shown to have a similar effect on B cells, since T cell-independent activation of B cells in the presence of CBD resulted in decreased IL-10 production; however, in the case of T cell-dependent activation, it was increased (71). We saw opposite effects to what we described in THP-1 macrophages treated with CBD (5 μM), since LPS-induced inflammatory cytokine (e.g., IL-6 and TNF-α) production was decreased (72), as was IL-1β secretion in the case of LPS-nigericin-induced activation (73). In PBMC-derived monocytes CBD once again decreased the secretion of IL-1β and IL-6 induced by activation of most TLRs (i.e., TLR1-9, with the exception of TLR3 and -8 for IL-1β and TLR1 and -3 for IL-6) (61). In contrast, in CD4^+^ or CD8^+^ T cells CBD did not influence LPS-induced cytokine production (50), underpinning the point that the effect of pCBs cannot be generalized among different cell types.

One of the most important functions of moDCs is the activation of naïve T cells, which allows them to act as a link between the innate and adaptive arms of the immune system. moDCs differentiated in the presence of CBD showed comparable T cell activation capabilities to vehicle-treated cells, but decreased the capability of LPS-activated moDCs to do the same (**Figure 6A**).

The mixed and sometimes contradictory results obtained with CBD treatment can be attributed to multiple factors. On one hand, CBD can influence many signaling pathways, both by direct binding to ionotropic and metabotropic as well as nuclear receptors, and by less direct actions on enzymes and transporters (36), and no single receptor has yet been identified that could have such wide-ranging effects (74). In light of this molecular promiscuity of CBD, we wanted to investigate its effects in a more detailed way by bulk RNA-Seq and subsequent pathway analysis. Despite the minimal effect on viability and cell surface markers as detailed above (**Figures 1**, **2**) we found that CBD induced several proinflammatory pathways, although not as significantly as LPS treatment applied alone (**Figures 5A, B**).

Notably this proinflammatory effect was not additive between the two treatments, as co-application of CBD and LPS resulted in the activation of IL-10 signaling, a markedly anti-inflammatory pathway as well as a shift towards Th2 signaling in moDCs (**Figure 5C**). In macrophages, CBD treatment results in the activation of similar pathways, as both the “Response to type I interferon” and “Response to metal ion” pathways were found to be upregulated, although the former to a lesser extent than in our results (75). CBD has a more clearly anti-inflammatory effect in other models, since it downregulates inflammasome activation and pyroptosis in oral keratinocytes (76) and depletes macrophages and promotes the development of myeloid-derived suppressor cells and DCs in mice (77). In LPS-stimulated RAW264.7 macrophages CBD induced an anti-inflammatory effect comparable to dexamethasone, as both reduced LPS-induced NO, IL-6, and TNF-α levels (62).

The shift in moDCs towards Th2 signaling did not translate into Th2 polarization of cocultured T cells. We observed a Th2 shift in LPS treated cells in our cocultures **(Figures 6B–D)** most likely due to the relatively low dose we applied (250 ng/ml) which might not be sufficient to push the T cell polarization toward a more inflammatory phenotype. This effect was abrogated in CBD-moDCs (**Figures 6B–D**), consistent and most likely due to their increased IL-10 production (**Figure 4E**), as IL-10 has been reported to have autocrine effects on DCs. These include inhibition of their trafficking to lymph nodes as well as their cytokine and chemokine production, and can lead to the failure to recruit and induce differentiation of naïve T cells (78, 79).

This supports the shift of CBD+LPS-treated cells towards a rather tolerogenic, IL-10 secreting phenotype. The decreased T cell proliferation is most likely due to the actions of IL-10, as we observed no increase of indoleamine-2,3-dioxygenase expression, which would decrease the availability of tryptophan and lead to subsequent immunosuppressive microenvironment (80). Previous reports have shown that both CBD and THC can stimulate the suppression of tryptophan degradation via this enzyme in THP1 cells, effectively increasing the availability of tryptophan (81). It is also in contrast to the effect of THC, since moDCs differentiated in the presence of the major psychotropic cannabinoid caused a lower percentage of proliferating T cells in a mixed leukocyte reaction as compared to control cells. Notably, THC also decreased the T cell activation caused by activated moDCs, which mirrors our results on LPS-activated CBD-treated moDCs (40). Importantly, although CBD acting directly on T cells can induce Treg responses (82), and has generally immunosuppressive effects by downregulating CD25 expression (83), in our experiments CBD was used only during the differentiation of moDCs, and was not applied during moDC-T cell cocultures. This also highlights the fact that care should be taken when administering CBD to cancer patients, who can receive cannabinoids to help stimulate their appetite (84), since its immunosuppressive effects (34) can help tumors escape immune surveillance, and can also interfere with immune checkpoint therapy (85).

CBD can also impact the endocannabinoid system in these cells, which might show different levels of activity between donors, and could result in the relatively high donor-dependence of some of the observed effects. CBD can influence classical cannabinoid receptors (either as an activator or an inverse agonist/antagonist, depending on the molecular context), and the endocannabinoid tone via its effect on the synthesizing and degrading enzymes of endocannabinoids (86–88). The endocannabinoid system is present in human DCs (89, 90), and these cells are capable of producing endogenous ligands such as 2-arachidonoylglycerol and N-arachidonylethanolamine (2-AG and AEA), especially during their activation (91). These endocannabinoids themselves can have both pro- and anti-inflammatory effects on immune cells, depending on numerous factors.

AEA modulates the function of macrophages and monocytes in a dose- and time-dependent manner. In the case of human PBMCs AEA decreased IL-6 and CXCL8 production at low concentrations, and fully suppressed TNF-α, IFN-γ and IL-4 secretion at higher concentration (92). On J774 macrophages AEA suppressed LPS-induced NO and IL-6 level in a concentration-dependent manner, while 2-AG decreased IL-6 production, but elevated iNOS-mediated NO production (93). On human DCs and plasmacytoid DCs AEA significantly blocked TLR7/8-induced IL-12 and IL-6 production in the former and IFN-α by the latter (94).

2-AG, the other major endocannabinoid, increased NO production from monocytes, as well as decreasing their motility through CB_1_ (95). In macrophages 2-AG decreases LPS-induced cytokine production and NO synthesis, although these effects are most likely not directly due to receptors activated by it. Rather it is the metabolite of 2-AG generated by cyclooxygenase-2, prostaglandin D2-glycerol ester that underlies the markedly anti-inflammatory effects (96).

Cyclooxygenases can also be influenced by CBD and other pCBs, as a mix of CBD, (-)-cannabigerol and THCV has been found to act synergistically with non-steroidal anti-inflammatory drugs to reduce CXCL8 expression in macrophages and lung epithelial cells. The combination of the pCBs with diclofenac also had a reducing effect on COX-1 and COX-2 gene expression and decreased IL-6, CXCL8 and CCL2 levels, which once again underlines the complex regulatory effect that CBD can have (97).

Based on these encouraging results human studies have also been initiated, as well as a few clinical trials specifically with CBD. As expected, not all the beneficial effects are due to direct action on immune cells, and the mechanism of action is generally difficult to pinpoint.

In the skin CBD decreased metalloproteinase activity in UVB-irradiated keratinocytes from psoriatic patients and normalized the expression of angiogenetic factors (98).

A randomized single center-controlled trial tested the effect of topical CBD for the treatment of thumb basal joint arthritis, and showed improvements of the visual analog scale pain, disabilities of the arm, shoulder, hand, and single assessment numeric evaluation scores (99).

Furthermore, CBD can elicit anti-inflammatory effects in an allergic contact dermatitis model, where CBD treatment resulted in an elevation in the levels of AEA, and also blocked the increase of IL-6, CXCL8, TNF-α, MCP-2 in polyinosinic-polycytidylic acid-stimulated HaCaT keratinocytes (100).

One possible mechanism behind these varied and sometimes contradictory effects of CBD is its effect on cholesterol homeostasis. In multiple human cell lines CBD has been shown to incorporate into cellular membranes, alter cholesterol accessibility, and disrupt cholesterol-dependent membrane properties (101). While this is not sufficient to explain all observed effects, it further broadens the potential targets of CBD.

Overall, our results point to the importance of cell-specific effects of pCBs, and to the fact that an integrated overview of their effect is required to accurately predict their effect on complex immunological processes. The results presented in this manuscript show that CBD can boost the secretion of proinflammatory cytokines *in situ*, but overall dampens adaptive responses as shown by their increased IL-10 secretion and the concomitant decrease in T cell proliferation.

## Data availability statement

The datasets generated for this study can be found in the Gene Expression Omnibus repository hosted at the National Library of Medicine, under accession number GSE235310.

## Ethics statement

The studies involving humans were approved by Regional and Institutional Ethics Committee of the Faculty of Medicine at the University of Debrecen (Debrecen, Hungary; approval number: OVSZK 3572-2/2015/5200). The studies were conducted in accordance with the local legislation and institutional requirements. The human samples used in this study were acquired from a by- product of routine care or industry. Written informed consent for participation was not required from the participants or the participants’ legal guardians/next of kin in accordance with the national legislation and institutional requirements.

## Author contributions

AS and ZP designed the study, ZP performed the experiments, analyzed the data, and prepared the figures. AS and ZP wrote the manuscript. AO provided material support. SA and DH provided research input and contributed to data interpretation. SA, DH, AO, BT, and AB contributed to manuscript editing. AS supervised the project. All authors contributed to the article and approved the submitted version.
