# Supplementary material for: The dual role of cannabidiol on monocyte-derived dendritic cell differentiation and maturation
Source: Front Immunol. 2023 Aug 22;14:1240800. doi: 10.3389/fimmu.2023.1240800 (PMC10482398; doi:10.3389/fimmu.2023.1240800)
Supplement: Supplementary file 1 [file DataSheet_1.docx]

Supplementary Material

## Supplementary Figures

**Supplementary Figure 1.** Changes in differentiation and maturation markers between monocytes and moDCs. Monocytes were stained directly after magnetic separation (mono) or cultured in the presence of GM-CSF and IL-4 for five days to generate moDCs in the presence of 0.1 v/v% absolute ethanol. Percentage of cells positive for CD14 (**A**), DC-SIGN (**B**), CD1a (**C**), HLA-DQ (**D**), CD83 (**E**) CCR7 (**F**) and CD86 (**G**). N=5, mean±SD, *p < 0.05, **p < 0.01, ***p < 0.001, ****p < 0.0001 as indicated (determined by paired T-test). Individual donors are represented by symbols.

**Supplementary Figure 2.** Pairwise comparison of CD86, HLA-DQ and CCR7 expression between vehicle- and CBD-treated moDCs. Monocytes were cultured in the presence of GM-CSF and IL-4 for five days to generate moDCs in the presence of 10 µM CBD or vehicle (0.1 v/v% absolute ethanol). Percentage of cells positive for CD86 (**A**), HLA-DQ (**B**), and CCR7 (**C**). N=11-14 donors, mean±SD. Individual donors in each treatment group are represented by symbols. Connecting lines mark cells from the same donor. CBD: (‑)-cannabidiol

**Supplementary Figure 3.** CBD-treated moDCs show minimally lower HLA-DQ expression after TLR ligand challenge compared to vehicle-treated cells. Monocytes were cultured in the presence of GM-CSF and IL-4 for five days to generate moDCs in the presence 10 µM CBD or vehicle (0.1 v/v% absolute ethanol). Maturation was induced by lipopolysaccharide (LPS: 250 ng/ml) and TLR7/8 activation (CL075: 500 ng/ml, both for 24 hours on day 5). Mean fluorescence intensity (MFI) of HLA-DQ staining is shown. N=6 donors, mean±SD, *p < 0.05, **p < 0.01 by repeated measures one-way ANOVA, as indicated. Individual donors are represented by symbols. CBD: (‑)-cannabidiol, CL075: TLR7/8 agonist, LPS: lipopolysaccharide.

**Supplementary Figure 4.** Acute CBD treatment increases IL-10 and TNFα cytokine production induced by TLR4 and TLR7/8 activation on moDCs, while decreasing the production of IL-6. Monocytes were cultured in the presence of GM-CSF and IL-4 for five days to generate moDCs. CBD or vehicle (0.1 v/v% absolute ethanol) treatment was applied on day 5, 10 min prior to lipopolysaccharide (LPS) treatment. Maturation was induced by applying (LPS: 250 ng/ml) for 24 h, also on day 5. IL-6 (**A**), IL-10 (**B**), and TNFα (**C**) production was determined with ELISA from supernatants. Bar plots represent mean ± SD of representative results from N=3 independent experiments, ** p < 0.01, ***p < 0.001, **** p<0.0001 compared to the marked groups as determined by repeated measures one-way ANOVA. ND, not determined. CBD: (‑)-cannabidiol, LPS: lipopolysaccharide.

**Supplementary Figure 5.** Representative flow cytometry dot plots and histograms showing forward and side scatter (FSC, SSC) as well as CFSE fluorescence intensity in moDC-T cell cocultures. Monocytes were cultured in the presence of GM-CSF and IL-4 for five days to generate immature moDCs in the presence of 10 µM concentration of CBD or vehicle (0.1 v/v% absolute ethanol). Maturation was induced by applying lipopolysaccharide (LPS: 250 ng/ml) treatment for 24 h on day 5. Percentage of proliferating T cells after 5 days of coculture of naïve T cells and moDCs at a ratio of 10:1 is shown by CFSE+ marker on histograms. CBD: (‑)-cannabidiol, LPS: lipopolysaccharide.

**Supplementary Figure 6.** CBD treated, LPS stimulated moDCs were less effective at inducing Th2 polarization of T cells. Monocytes were cultured in the presence of GM-CSF and IL-4 for five days to generate immature moDCs in the presence of 10 µM concentration of CBD or vehicle (0.1 v/v% absolute ethanol). Maturation was induced by applying lipopolysaccharide (LPS: 250 ng/ml) treatment for 24 h on day 5. Percentage of IL-4^+^ T cells after 5 days of coculture of naïve T cells and moDCs at a ratio of 10:1. N=3, individual donors are represented by symbols. Connecting lines mark cells from the same donor. CBD: (‑)-cannabidiol, LPS: lipopolysaccharide

**Supplementary Figure 7.** Non-psychotropic pCBs do not influence LPS and CL075-induced maturation of moDCs. Monocytes were cultured in the presence of GM-CSF and IL-4 for five days to generate moDCs in the presence 10 µM CBN, THCV or vehicle (0.1 v/v% absolute ethanol). Maturation was induced by lipopolysaccharide (LPS: 250 ng/ml) and TLR7/8 activation (CL075: 500 ng/ml for 24 hours on day 5). Percentage of cells positive for DC-SIGN (**A**), CD83 (**B**), CD86 (**C**), HLA-DQ (**D**), CD1a (**E**), and CCR7 (**F**) are shown. N=6-9 donors, mean±SD. Individual donors are represented by symbols. CBN: (‑)-cannabinol, THCV: (‑)-Δ^9^-tetrahydrocannabivarin, CL075: TLR7/8 agonist, LPS: lipopolysaccharide.
